# Meiotic Genes and DNA Double Strand Break Repair in Cancer

**DOI:** 10.3389/fgene.2022.831620

**Published:** 2022-02-18

**Authors:** Lea Lingg, Sven Rottenberg, Paola Francica

**Affiliations:** ^1^ Institute of Animal Pathology, Vetsuisse Faculty, University of Bern, Bern, Switzerland; ^2^ Cancer Therapy Resistance Cluster, Department for BioMedical Research, University of Bern, Bern, Switzerland; ^3^ Bern Center for Precision Medicine, University of Bern, Bern, Switzerland

**Keywords:** meiosis, mitosis, meiotic genes, genomic instability, DNA repair, homologous recombination

## Abstract

Tumor cells show widespread genetic alterations that change the expression of genes driving tumor progression, including genes that maintain genomic integrity. In recent years, it has become clear that tumors frequently reactivate genes whose expression is typically restricted to germ cells. As germ cells have specialized pathways to facilitate the exchange of genetic information between homologous chromosomes, their aberrant regulation influences how cancer cells repair DNA double strand breaks (DSB). This drives genomic instability and affects the response of tumor cells to anticancer therapies. Since meiotic genes are usually transcriptionally repressed in somatic cells of healthy tissues, targeting aberrantly expressed meiotic genes may provide a unique opportunity to specifically kill cancer cells whilst sparing the non-transformed somatic cells. In this review, we highlight meiotic genes that have been reported to affect DSB repair in cancers derived from somatic cells. A better understanding of their mechanistic role in the context of homology-directed DNA repair in somatic cancers may provide useful insights to find novel vulnerabilities that can be targeted.

## Introduction

In the 19th century, the hypothesis was put forward that cancers arise from embryonic remnants that remain in adult organs (Durante, 1874; Cohnheim, 1875). When the microenvironment changes and provides the necessary blood supply, these remnants grow in an uncontrolled fashion. This theory was based on the observation of pathologists that the microscopic morphology of some cancers (*e.g.* Teratoma and Wilm’s tumor) highly resembles that of embryonic tissues. Later on, Theodor Boveri (1862–1915) concluded from his observations that embryonic characteristics of cancer cells are rather side effects of the abnormal distribution of chromosomes and that remnant embryonic tissues only explain rare cases (Boveri, 2008). This fostered the concept that cancer cells can arise from well-differentiated cells and can de-differentiate. Today we know that cancer is caused by various genetic alterations that affect both germ cells and somatic cells. Intriguingly, many somatic cancer cells seem to benefit from the expression of genes that are typically present in germ cells and contribute to meiotic cell division. As several of these affect processing of DNA double strand breaks in the context of homologous recombination, some cancers may benefit from the double-strand break (DSB) repair mediated by aberrantly expressed meiotic genes. If particular cancers are dependent on their expression when exposed to DNA damage, they may provide interesting drug targets. Whereas normal somatic cells do not depend on the expression of meiotic genes for DSB repair, tumor cells that do depend on them in the context of DNA damage may die when their function is blocked. Such a therapeutic approach would still harm germ cells, but since many cancers arise in people beyond the wish to have children, the loss of germ cells may be tolerated. In this review, we briefly highlight mitotic and meiotic cell division with a focus on DSB-related meiotic genes that have been found to be aberrantly expressed in cancer.

**FIGURE 1:**
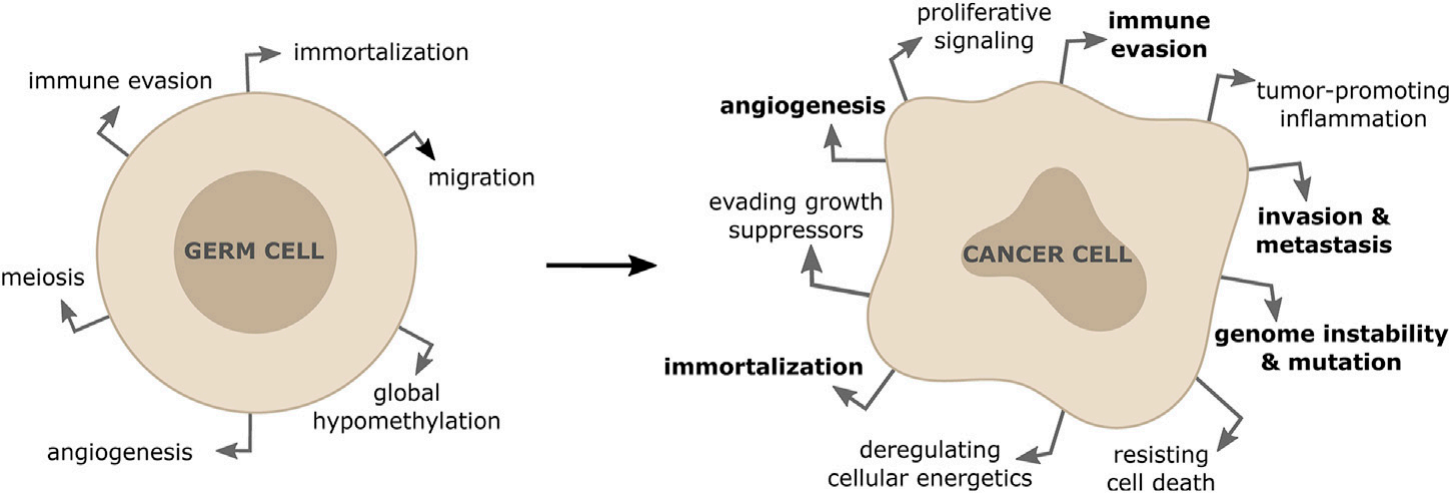
Activation of meiotic genes in somatic cells contribute to properties of tumor formation and progression. The re-expression of meiotic genes in somatic cancer cells is responsible for driving some of the hallmarks of cancers (shown in bold).

The primary goal of each cell division for non-cancerous somatic cells is to ensure that daughter cells are genetically identical to their parent cells (Nurse, 2000). Errors happening during cell division result in various forms of genome alterations in the daughter cells and include mutations of specific genes, amplifications, deletions or rearrangements (including gain or loss) of entire chromosomes (Levine and Holland, 2018). Cells use a number of mechanisms to prevent these alterations, including error-free repair of sporadic DNA damage, high fidelity DNA replication during S-phase, precise chromosome segregation during mitosis and a coordinated cell cycle progression (Shen, 2011). Inherited or acquired defects in DNA repair, DNA replication, chromosome segregation or cell cycle control lead to an increased mutation frequency. Accumulation of these genomic alterations is generally referred to as genome instability, which predisposes cells to malignant transformation (Negrini et al., 2010). In most cases, significant genome alterations result in a non-viable cell, but in rare events it might confer a selective growth advantage, leading to cancer initiation and progression. It has been clear for a long time that such genomic changes involve genes encoding tumor suppressors, proto-oncogene or genes that function to maintain genomic integrity (Negrini et al., 2010). Moreover, there is emerging evidence that an inappropriate activation of meiotic genes in somatic cells results in both initiation and maintenance of the malignant phenotype in a range of cancer types (Feichtinger and McFarlane, 2019). The aberrant expression of meiotic genes in cancer cells has been shown to contribute to various hallmarks of cancer by altering centromeric polarity control, motility, chromosome dynamics and DNA repair (Hanahan and Weinberg, 2011; McFarlane and Wakeman, 2017) (Figure 1). In particular, alterations of how cancer cells repair DNA breaks due to unscheduled expression of meiotic genes, has been shown to drive genomic instability and to affect tumor cells’ response to anticancer therapies (Nielsen and Gjerstorff, 2016; Mantere et al., 2017; Trussart et al., 2018).

These observations have raised a significant interest towards the study of meiotic genes in somatic cancers, as they could be used as cancer-specific predictive biomarkers of therapy response. Moreover, in the era of immunotherapy aberrantly expressed germ cell proteins are prime targets for cancer vaccination and adoptive T-cell transfer with chimeric T-cell receptors. For example, male germ cells lack HLA-class I molecules and cannot present antigens to T cells to induce immunotolerance (Janitz et al., 1994). When expressed in somatic cancers, cancer/testis antigens therefore represent promising targets for cancer immunotherapy (Gjerstorff et al., 2015).

## Mitosis and Meiosis

Eukaryotic cells can undergo two different types of cell divisions. On the one hand, with the goal of maintaining a functional organism, somatic cells undergo mitosis and thereby create two genetically identical daughter cells (Nurse, 2000). On the other hand, germline cells undergo a different type of cell division, known as meiosis, to produce haploid gametes, which have only one copy of each chromosome. Both processes are tightly regulated by a number of coordinated pathways to ensure the correct segregation of genetic material. The molecular mechanisms of mitosis and meiosis are well described in other reviews (Nurse, 2000; Marston and Amon, 2004; Duro and Marston, 2015; Ohkura, 2015; Bolcun-Filas and Handel, 2018); we therefore provide only a succinct overview of both processes here.

### Mitosis

In brief, cells undergo four different phases during the cell cycle: the two main phases, S- and M-phase (mitosis), are separated by two gap phases called G1 (before S-phase) and G2 (after S-phase). To create two identical daughter cells from a parental cell, chromosomes are duplicated during S-phase to form sister chromatids, which will be separated to each daughter cell in the M-phase of the cell cycle. G1 and G2 are important to provide cells time to control the correct replication and chromosomal segregation (Nurse, 2000; Williams and Stoeber, 2012). The transition from one phase to another is tightly regulated by cyclin-dependent kinases (CDKs), which phosphorylate downstream factors allowing cells to initiate DNA replication or chromosomal segregation to the daughter cells (Barnum and O’Connell, 2014).

### Meiosis

Meiosis is cell division for the generation of gametes in sexual reproduction. The key feature of this process is the reduction of the DNA content with the final goal of generating gametes with a haploid set of DNA. This process involves two cycles of cell division: meiosis I and meiosis II. In meiosis I, homologous chromosomes are replicated and subsequently segregated, generating diploid daughter cells. Meiosis I is followed by another round of chromosome-segregation (Meiosis II), which does not include another phase of DNA replication and gives rise to four haploid gametes. Gametes originating from the same cell are genetically different from each other, not only due to the independent segregation of maternal and paternal DNA but also due to another mechanism exclusive to meiosis I: before segregation of the homologous chromosome pairs in meiosis I, chromosomes undergo a programmed recombination of the genetic material, also known as homologous recombination (HR), which involves the formation of several DSBs. The repair of these lesions is associated with non-crossover or crossover events, which in the latter case leads to the exchange of genetic information and thus to an increase in inter-individual diversity (Ohkura, 2015; Bolcun-Filas and Handel, 2018).

**FIGURE 2:**
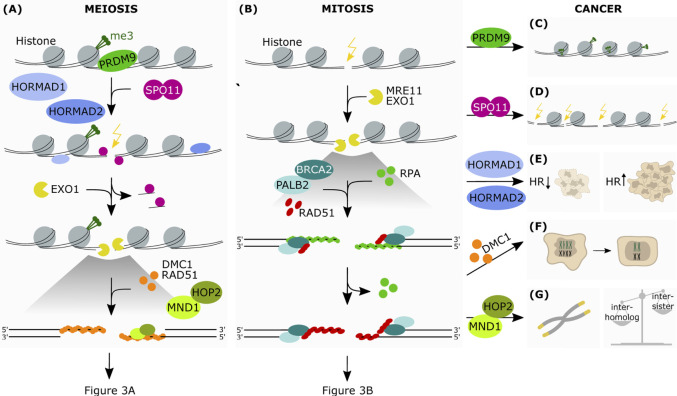
The aberrant expression of meiotic genes in somatic cancers affects HR-dependent-DNA repair. Simplified schematic representation of the first steps of HR in meiosis **(A)** and mitosis **(B) (A)** PRDM9 is the protein responsible for the epigenetic marking of the hotspots for DSB introduction. This allows the binding of SPO11, which is favoured by HORMAD1 and HORMAD2. SPO11 introduces strand breaks at the marked hotspots. This is followed by end resection at the break sites by exonucleolytic activity. The subsequent binding of the RAD51 and DMC1 recombinases onto ssDNA allows the formation of a nucleoprotein filament, which in turn recruits downstream factors promoting interaction between homologous chromosomes. The protein heterodimer HOP2-MND1 acts in concert during this process favoring homology search and therefore resolution of the DSB **(B)** Upon recognition of the break site, the nucleases MRE11 and EXO1 resect the DNA generating ssDNA which are stabilized by RPA. This allows the formation of the RAD51-ssDNA filaments, in cooperation with BRCA2 and PALB2, which search for the homologous DNA template by invading the sister chromatid **(C)** In some cancer cells PRDM9 may also mark DNA regions that are favorable to the formation of chromosomic lesions **(D)** Due to its meiotic function in generating DNA strand breaks, aberrantly expressed SPO11 may then promote crossover events in somatic cancer cells, as well as translocations, insertions and deletions **(E)** Due to its ability in modulating HR-mediated DNA damage repair, increased expression of HORMAD in somatic cancers has been shown to promote or disrupt HR-mediated repair, depending on the genetic background **(F)** Expression of DMC1 promotes meiosis-like reductional segregation of homologues in polyploid cells, restoring the proliferative state of somatic cancer cells **(G)** HOP2-MND1 may function in cancer cells to promote an alternative lengthening of telomeres (ALT) in the absence of telomerase reactivation. Furthermore, as HOP2-MND1 favor recombination between homologous chromatids in meiotic cells, their reactivation in somatic cancer cells could disrupt the recombination bias between sister chromatids that is typical of mitotic cells.

**FIGURE 3:**
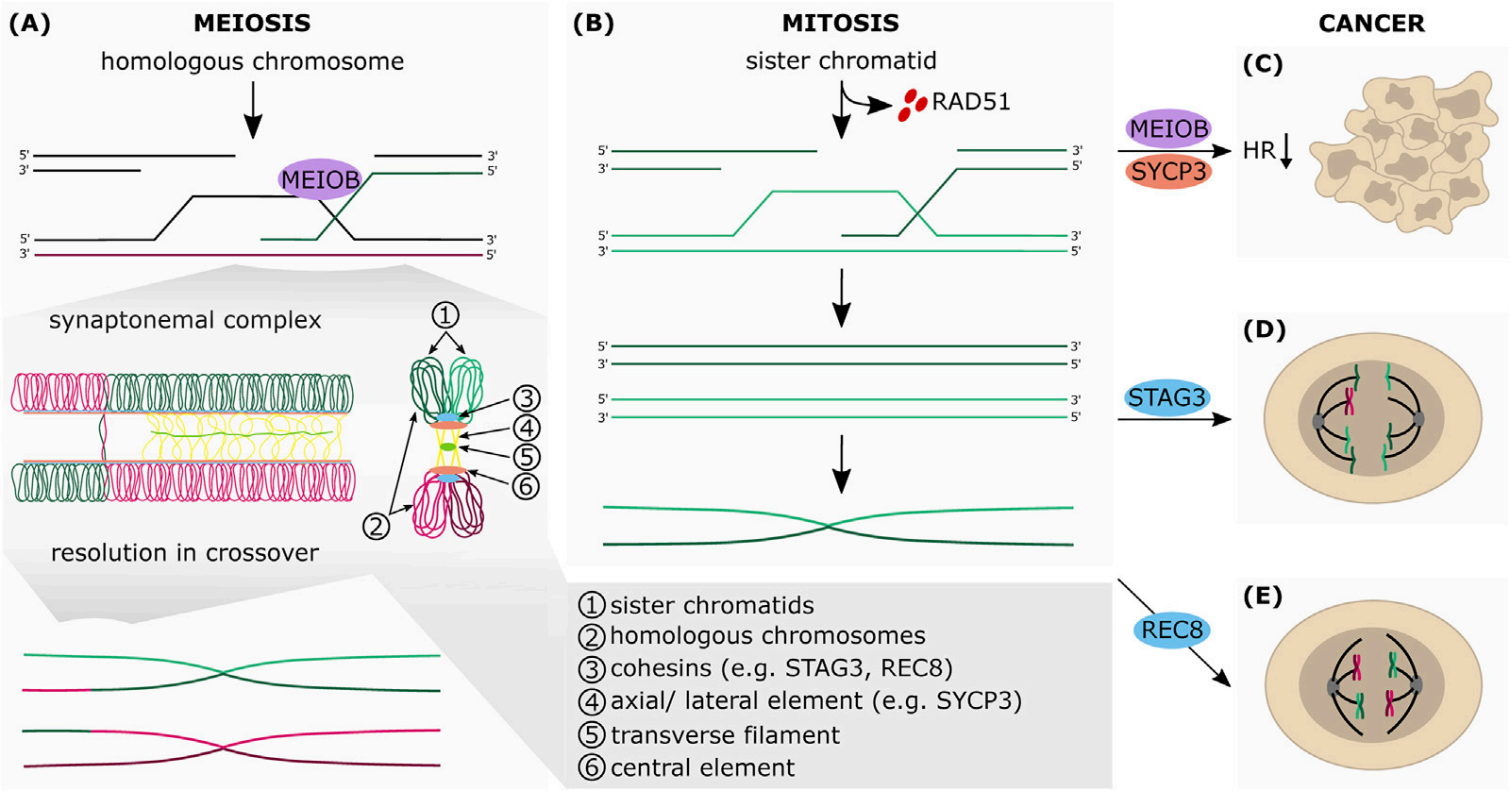
The aberrant expression of meiotic genes in somatic cancers affects HR-dependent-DNA repair. Simplified schematic representation of the final steps of HR in meiosis **(A)** and mitosis **(B) (A)** During meiosis, the sister chromatids (1) are connected with each other by the cohesin complex (3). Cohesins are also essential for the formation of the synaptonemal complex (SC), which connects the homologous chromosomes (2). The SC consists of the axial elements (AEs) of the chromosomes (4), which are connected to each other by transverse filaments (5) and the central element (6). Upon formation of the SC, AEs are turned into lateral elements (LEs), which consist of two different SC proteins: SYCP2 and SYCP3. Between the AEs of the two homologous chromosomes SYCP1 builds parallel dimers that stabilize the positioning of the homologous chromosomes and favor crossovers. The exonucleolytic activity of MEIOB allows the formation of a double Holliday junction between two homologous chromosomes which can be resolved in both non-crossover and crossover formation **(B)** The RAD51-ssDNA filaments search for homologous DNA template by invading the sister chromatid, which leads to the formation of the Holliday junctions and finally to DNA synthesis of the missing sequence that was lost at the break point. After synthesis, the junctions are resolved by endonucleolytic cleavage, the invading strand is released and anneals to the other side of the break. The repair is completed by ligation of the gaps by a DNA ligase **(C)** Aberrant expression of MEIOB and SYCP3 in somatic cancers inhibits HR-mediated DNA repair **(D)** STAG3 altered expression mediates chromosomal mis-segregation and genome reduction of cancer cells **(E)** Augmented expression of REC8 in somatic cancer cells promotes meiosis-like reductional segregation of homologous chromosome, which provides a survival advantage following DNA damage-inducing treatment.

In contrast to the programmed generation and repair of DSBs during meiotic cell division, DNA lesions occur randomly in somatic cells, and need to be repaired in an error-free manner to minimize the risk of DNA alterations. To this purpose, somatic cells also use HR, which repairs DSBs with high fidelity. HR in somatic cells is restricted to S- or G2 -phase of the cell cycle as it relies on the presence of a sister chromatid as a template for DNA repair, though the homologous chromosome can also be used as a template with a much lower frequency (Kadyk and Hartwell, 1992; Takata et al., 1998). HR in somatic cells is very well described (Li and Heyer, 2008; Wright et al., 2018; Scully et al., 2019) and will therefore not be discussed in further details in this review. Overall, HR in mitosis and meiosis share many similarities, but they do involve different key players (Figures 2, 3). In this review we will describe in more detail the process of HR in human meiotic cells.

## HR in Meiotic Cells

### Marking of Hotspot Sites and Introduction of DSBs

In contrast to mitosis, DSBs in meiotic cells are introduced in a programmed way and many factors involved in the repair are uniquely expressed in meiosis. The program is initiated at recombination hotspots, which are preferentially targeted for DSB formation. In mice and humans, PRDM9 is the main protein catalyzing the epigenetic marking and thus the initiation of the break-inducing process (Tock and Henderson, 2018). The PRDM9 zinc-finger domain is able to bind specific DNA sequences, bringing the PR/SET domain in position to allow trimethylation of histone H3 on lysine 4 (H3K4me3) and histone H3 on lysine 36 (H3K36me3) (Parvanov et al., 2010; Grey et al., 2011; Powers et al., 2016). Epigenetic modifications of H3K4 are not only promoted by PRDM9, they are also commonly induced at promoters or enhancers by other methyltransferases (Brick et al., 2012; Baudat et al., 2013; Tock and Henderson, 2018). Therefore, it is not surprising that upon loss of PRDM9 DSBs are still introduced at PRDM9-independent H3K4me3 sites, even though they result in inefficient repair and meiotic arrest (Berg et al., 2010; Brick et al., 2012). These findings show that trimethylation of H3K4 is not sufficient to induce a successful recombination, even though the exact mechanism remains elusive (Baudat et al., 2013). In a next step, a DSB machinery consisting of SPO11, IHO1, MEI4, MEI1 and REC114 needs to be activated. These members are evolutionarily conserved among eukaryotes (Kumar et al., 2010; Baudat et al., 2013). Besides SPO11, which is the catalytically active unit, IHO1, MEI4, MEI1 and REC114 are crucial for the introduction of DSBs and the preferential interaction with the homologous chromosome instead of the sister chromatid (Libby et al., 2003; Kumar et al., 2010; Stanzione et al., 2016; Kumar et al., 2018). SPO11 is highly conserved among eukaryotes, suggesting an important role of this protein in meiotic DSB repair. It is responsible for the introduction of the strand break at the marked hotspots by performing a topoisomerase-like reaction: its tyrosine residue attacks a phosphorous on the DNA, which then triggers the formation of a tyrosyl phosphodiester linked to DNA. This in turn disrupts the double-helix and introduces a DNA break (Keeney et al., 1997).

### DNA end resection and initiation of the synaptonemal complex

To allow further processing of the DNA break site, degradation of the 5′ end is required. End resection occurs by a two-step mechanism. In a first step, CtIP activates the Mre11-Rad50-Nbs1 (MRN) complex to endonucleolytically cleave the 5′- terminated DNA strands close to where SPO11 is bound. This in turn, releases SPO11 with short oligonucleotides from the DNA ends bound to it (Neale et al., 2005; Keeney, 2008; Garcia et al., 2011; Symington, 2016). In a second step, EXO1 and/or DNA2 nucleases extend the resected tracts to produce long 3′-ssDNA overhangs, which favors homology search (Symington, 2016). While in prokaryotes RecA is the only protein involved in homology search and strand invasion, in eukaryotes two of its homologs are involved: RAD51, which is also active in mitotic HR, and DMC1, which is exclusively expressed in meiotic cells (Bugreev et al., 2011). Similarly to the process in somatic cells, BRCA2 is required for proper loading of DMC1 and RAD51, since BRCA2-deficient spermatocytes can induce DSBs but fail in completing recombination (Sharan et al., 2004). Successful binding of DMC1 proteins onto ssDNA allows the formation of a nucleoprotein filament (Sehorn et al., 2004), which in turn promotes the interaction between homologous chromosomes. This process was shown to be stimulated by five Rad51 paralogs (RAD51B, RAD51C, RAD51D, XRCC2 and XRCC3), which prime the nucleoprotein filaments for strand exchange with the template duplex (Taylor et al., 2015). Moreover, the protein heterodimer HOP2-MND1 acts in concert during this process favoring homology search and therefore resolution of the DSB (Tsubouchi and Roeder, 2002; Chen et al., 2004). Besides recruiting MND1 to the break sites, HOP2 favors the interaction between homologous chromosomes over sister chromatids (Leu et al., 1998). There are three main modes of action of the HOP2-MND1 complex. It orchestrates the localization of DMC1 on the ssDNA and stabilizes the nucleoprotein complex (1) (Pezza et al., 2007). This allows DMC1 to induce the formation of a D-loop and the synaptonemal complex (SC) and together with HOP2-MND1, it brings homologs in close juxtaposition (2) (Chen et al., 2004; Pezza et al., 2007). Finally, HOP2-MND1 enhances the homology search by the condensation of the dsDNA around the filament (3) (Pezza et al., 2010).

### Sister Chromatid Cohesion

The role of cohesins is crucial for the next steps of meiosis. Cohesion is not specific to meiosis but also occurs during mitosis and is essential for DNA replication, DNA repair, gene expression and development (Brooker and Berkowitz, 2014). During meiosis I, sister chromatids associate with each other via cohesins along the chromatid arms and at the centromere. The meiosis-specific members of this complex are SMC1β, REC8, RAD21L and STAG3, while SMC1α, RAD21 and STAG2 have been reported to be active in germ cells as well as in somatic cells (Brooker and Berkowitz, 2014). The chiasmata formed upon HR links the homologous chromosomes and allows their localization in the metaphase plate. This specific localization of the chromosomes triggers the attachment of the microtubules from the spindle machinery in a syntelic manner: the sister kinetochores of the maternal centromeres are attached to microtubules with opposite orientation of the paternal centromeres (Peters et al., 2008; Brooker and Berkowitz, 2014; Ishiguro, 2019). Segregation of the homologous chromosomes in meiosis I is triggered by the cleavage of the cohesins along the sister chromatid arms and the resolution of the chiasmata. A crucial component of the meiotic cohesin complex is REC8. The separase enzyme cleaves REC8 only from the sister chromatid arms, leaving the cohesins at the centromeres (Marston and Amon, 2004). REC8 knockout mice are sterile and show SC-like formation between sister chromatids instead of the homologous chromosomes (Xu et al., 2005). These data are further supported by the finding that cohesin at centromeres influences the orientation of the kinetochores (Ogushi et al., 2021). This suggests that functional REC8 is crucial for HR and proper chromosome segregation in meiotic cells. How exactly cohesion at centromeres differs from the arm-cohesion remains to be elucidated.

### Synaptonemal Complex: Formation and Resolution

Cohesins are important for the formation of the SC as they initiate the recruitment of the complex members. The SC consist of a tripartite proteinaceous structure that is able to hold homologous chromosomes in close juxtaposition and allows formation of synapsis (Figure 3A). The SC includes three different parts: 1) the axial elements (AEs), that are assembled along the cohesin on the sister chromatid arms, and are connected to each other by transverse filaments (TFs) (2) and the central element (CE) (3) (Page and Hawley, 2004). Upon formation of the SC, AEs are turned into lateral elements (LEs). They consist of two different proteins SYCP2 and SYCP3 that form heterodimers (Yuan et al., 1998; Yang et al., 2006). Between the AEs of the two homologous chromosomes SYCP1 builds parallel dimers (TFs), which stabilize the positioning of the homologous chromosomes and favor crossovers (de Vries, 2005). The CE forms a network between SYCE1, SYCE2 and TEX12 that also interacts with the TF component SYCP1 (Yang and Wang, 2008). The SC controls in a feedback loop the generation of DSBs: the assembly of the central region triggers removal of HORMA-domain proteins which are essential for the recruitment of, for example, IHO1 and thus, hinders the assembly of the DSB machinery (Wojtasz et al., 2009; Hollingsworth, 2020; Mu et al., 2020). As mentioned earlier, after capturing the homologous chromosome that is close enough to the DSB site, the formation of the D-loop is triggered by DMC1. This structure can be either resolved as a non-crossover (NCO) or as a crossover (CO) after conversion into a double Holliday junction (dHJ). Following the D-loop formation and invasion of the ssDNA, the homologous non-sister chromatid can be used as a template to repair the break site. This process is defined as single-end invasion (Hunter and Kleckner, 2001). At this stage, the reannealing of the repaired end to its parental strand results in a NCO event but in some cases the D-loop is further processed into a dHJ if the second end of the DSB site is captured by the same homologous non-sister chromatid (Hunter and Kleckner, 2001; Petronczki et al., 2003). This complex structure can be resolved either as NCO, or CO if the cleavage is induced asymmetrically between the homologous chromosomes, thus generating reciprocal exchanges (Petronczki et al., 2003; Heyer, 2004). Luo et al. suggest that the conversion of the D-loop to dHJ and thus potential CO is dependent on MEIOB, which works in a complex with SPATA22 and RPA (Luo et al., 2013). Resolution of the dHJ is thought to be mediated by the resolvases MUS81-EME1, SLX1-SLX4 and GEN1 (triggering COs) or BLM (NCO dissolution) (Wyatt and West, 2014). Moreover, the presence of functional CE seems to be essential for successfully CO events (Baudat et al., 2013).

### Checkpoint Surveillance

The completion of meiosis I requires the coordination of different events. First of all, one CO event per homologous chromosome pair is inevitable. Second, the CO frequency has to be regulated and COs need to be evenly spaced along the chromatids. Overall, the interaction with homologous chromatids should be favored over sister chromatids (Baudat et al., 2013). One group of proteins that mainly serves as checkpoint controls in meiosis I are the HORMA (Hop1, Rev7, Mad2)-domain proteins HORMAD1 and HORMAD2. Loading of HORMAD1 was found to be initiated by REC8 and RAD21L, two members of the cohesin complex. Both HORMAD proteins cluster along AEs until the assembly of the SC, where they are removed and regulate DSB induction (Wojtasz et al., 2009; Fujiwara et al., 2020; Mu et al., 2020). While HORMAD1 plays a role in homology search by increasing the number of ssDNA ends as well as in the synaptonemal complex formation, HORMAD2 is exclusively responsible as a checkpoint control element (Shin et al., 2010; Daniel et al., 2011; Wojtasz et al., 2012). Shin et al. observed that in the absence of HORMAD1, more inter-sister chromatid repair takes place, suggesting that HORMAD1 promotes the use of homologous DNA over sister DNA for repair of DSBs (Shin et al., 2013). Furthermore, HORMAD1 recruits IHO1 to unsynapsed regions which in turn triggers DSB formation by SPO11 and its auxiliary proteins (Stanzione et al., 2016). Another control checkpoint at this stage is the detection of unsynapsed chromosomes. HORMAD2 seems to play a key role in this process. It recruits ATR kinases along unsynapsed axes and induces phosphorylation of H2AX (Turner et al., 2005; Wojtasz et al., 2012). This leads to meiotic silencing of unsynapsed chromatin as protecting mechanism (Turner et al., 2005).

Hence, to ensure the programmed crossover of genetic information during Meiosis I, germ cells express a toolkit of specific genes involved in the induction of DSBs and their repair.

## Meiotic DNA Repair Genes Aberrantly Expressed in Cancers

Intriguingly, several of these genes have been found to be aberrantly expressed in mitotic cancer cells, and they are thought to contribute to driving genomic instability and carcinogenesis. Here we describe the main genes involved (Figure 2 and Figure 3).

### HORMADs

HORMAD1 is one of the most studied meiotic genes implicated in carcinogenesis and genomic instability. In physiological conditions, HORMAD1 expression is restricted to meiotic cells in testes and ovaries. However, many studies have shown that HORMAD1 is significantly upregulated in several cancers where it correlates with increased genomic instability and poor patient prognosis (Adelaide et al., 2007; Watkins et al., 2015; Chen et al., 2018; Gao et al., 2018; Nichols et al., 2018; Gantchev et al., 2020). Increased expression of HORMAD1 has been detected in patient samples isolated from breast cancer (including triple-negative breast cancer (TNBC) and basal-like breast cancer (BLBC)) (Adelaide et al., 2007; Yao et al., 2014; Chen et al., 2018), lung cancer (lung adenocarcinoma (Yao et al., 2014; Nichols et al., 2018), lung squamous cell carcinoma (Yao et al., 2014), small cell lung cancer, NSCLC (Chen et al., 2005)), esophageal, endometrial, bladder, colon (Chen et al., 2005), epithelial ovarian carcinoma (Shahzad et al., 2013), gastric cancer (Aung et al., 2006), head and neck squamous cell carcinoma, melanoma (Yao et al., 2014) and cutaneous T-cell lymphoma (CTCL) (Tsang et al., 2018).

Recent studies have demonstrated that the positive correlation between increased HORMAD1 expression and genomic instability in tumors is due to its ability in modulating DNA damage repair (Watkins et al., 2015; Gao et al., 2018; Nichols et al., 2018; Liu et al., 2020). These studies suggest distinct hypothesis on how HORMAD1 affects HR-mediated DNA repair. The group of Andrew N. J. Tutt was the first to demonstrate that the positive correlation between HORMAD1 expression and chromosomal instability observed in TNBC is the consequence of the disruption of HR-mediated repair (Watkins et al., 2015). Using a panel of TNBC cell lines, as well as non-transformed cells, the authors showed that overexpression of HORMAD1 suppresses RAD51-dependent HR. This drives the error-prone 53BP1-dependent non-homologous end joining (NHEJ) DNA repair pathway. In addition, HORMAD1 expression correlated with a better response to HR defect-targeting agents (such as poly ADP-ribose polymerase inhibitors PARPi or poly ADP-ribose polymerase (PARP) inhibitors olaparib and BMN673) in both TNBC cell lines and clinical trial data. With their data, the authors provided a possible mechanism for the increased levels of allelic-imbalanced copy-number aberrations (AiCNA) that are abundant in TNBC.

In contrast, two distinct studies demonstrated that HORMAD1 promotes HR in models of lung adenocarcinomas, providing a selective survival advantage for cancer cells (Gao et al., 2018; Nichols et al., 2018). HORMAD1 loss enhanced sensitivity to irradiation (IR), camptothecin and PARP inhibition, and significantly reduced tumor growth *in vivo*. Mechanistically, Gao and colleagues showed that HORMAD1 re-distributes to nuclear foci and co-localizes with the DSB marker yH2AX in response to IR and chemotherapeutic agents (Gao et al., 2018). Both studies demonstrated that HORMAD1 expression promotes DSB repair by HR, thus offering a mechanistic explanation for the reduced sensitivity to the PARP inhibitor Rucaparib in the work of Wang and colleagues (Wang et al., 2018). The conflicting data on the modulation of HR by HORMAD1 reported in these studies (Watkins et al., 2015; Gao et al., 2018; Nichols et al., 2018), could be explained by the different cellular models that have been used. HORMAD1 might have opposing effects on HR in different cancers due to tissue-specific expression of HR pathway regulators targeted by HORMAD1. This may explain why HORMAD1 inhibits HR in TNBC and stimulates HR in lung adenocarcinomas.

More recently, HORMAD1 was shown to modulate another DNA repair pathway besides HR. The group of Yidan Liu showed that aberrant expression of HORMAD1 compromises DNA mismatch repair in cancer cells (Liu et al., 2007). Mechanistically, HORMAD1 interacts with the MCM8-MCM9 complex and prevents its efficient nuclear localization. Consequently, HORMAD1-expressing cancer cells have reduced MLH1 chromatin binding and DNA mismatch repair defects. HORMAD1 expression is also associated with an increased mutation load and genomic instability in a human cancer samples cohort from the TCGA dataset (Liu et al., 2020).

Even though the homologous protein HORMAD2 was found to be aberrantly expressed in lung cancer tissues (Liu et al., 2012), its potential role in modulating DNA repair in cancer cells is less clear. In one study, aimed at investigating the impact of candidate genes on thyroid carcinoma (THCA), the authors found that HORMAD2 was significantly hypermethylated in THCA cells. Treatment with the DNA hypomethylating agent 5-Azacitidine, suppressed THCA cells’ viability, motility and invasiveness (Lin et al., 2018). However, follow-up studies are needed to investigate a direct involvement of HORMAD2 in promoting cancer cell’ growth.

### HOP2-MND1

The group of Greenberg and colleagues discovered that the HOP2-MND1 heterodimer functions in cancer cells to promote an alternative lengthening of telomeres (ALT) mechanism in the absence of telomerase activity (Nam Woo Cho et al., 2014). Similar to meiotic recombination, this process involves the generation of DSBs to initiate the recombination between homologous DNA sequences on non-sister chromatids. Mechanistically, telomeres behave like a broken chromosome and serve as a substrate for DNA replication-dependent *de novo* telomere elongation, a process that is dependent on the ability of HOP2-MND1 to stimulate non-sister chromosome interactions (Nam Woo Cho et al., 2014). This discovery added a new class of factors to the mix of germline genes that become activated during oncogenesis. A role for HOP2 in tumors is also supported by several studies that have described *HOP2* germline mutations in familial breast and ovarian cancers (Peng et al., 2013a; Peng et al., 2013b; Yang et al., 2016). These mutations caused defective alternative splicing and truncated the open reading frame of the *HOP2* gene, generating an isoform that is expressed in the cytoplasm and it is often detected in tumor stromal cells. The splice variants act as dominant negatives to counteract wild type *HOP2* activity in transcription and to abolish Rad51 foci formation after IR-induced DNA damage. The constitutive expression of the HOP2 cytoplasmic isoform, but not the wild type, induced tumor growth in nude mice (Peng et al., 2013b). Another study from the same group found that mutant HOP2 protein production in the breast tumor microenvironment induced VEGF expression by enhancing VEGF promoter activity and potentially promote angiogenesis and adipogenesis (Yang et al., 2016). These results suggest that mutated HOP2 protein production in the tumor stroma may contribute to carcinogenesis and therefore could be used as a biomarker to define mutant reactive breast cancer stroma. *HOP2* mutations were also observed in cases of early onset familial breast and ovarian cancer and a *HOP2* mutation in the C-terminus (*HOP2 p.del201Glu*, is associated with XX ovarian dysgenesis (Zhao and Sung, 2015). Lastly, the group of I.V. Litvinov reported that the HOP2 protein is also ectopically expressed in cutaneous T-cell lymphomas (CTCL), suggesting that HOP2 expression is not unique to breast, ovarian and fallopian tube cancers (Tsang et al., 2018).

Although less is known about MND1 in carcinogenesis, its aberrant expression has been reported in ovarian cancers and lung adenocarcinoma (Yeganeh et al., 2017; Zhang et al., 2019; Wei et al., 2021; Zhang et al., 2021). By performing a differential mRNA expression analysis of normal versus malignant ovarian tumors, P.N. Yeganeh and colleagues identified *MND1* as one of the most significantly dysregulated genes in the malignant tissues (Yeganeh et al., 2017). In a recent study, genomic data from the GEO database that were further validated with clinicopathological data from the TCGA database revealed MND1 as a differentially expressed gene that significantly associated with overall survival of lung adenocarcinoma patients. The authors of the study therefore concluded that MND1 could be used as a prognostic biomarker and a molecular curative target for lung adenocarcinoma (Wei et al., 2021). However, in all these studies, the underlying molecular mechanism of how aberrant expression of MND1 contributes to carcinogenesis has not been reported. Using a genome-wide insertional mutagenesis screen in somatic cancer cells, we identified MND1 as a factor which increases cellular fitness following exposure to irradiation (IR) (Francica et al., 2020). Similarly, in somatic *Arabidopsis thaliana* cells, the homologue of MND1, AtMnd1, is induced by IR and its loss causes IR sensitivity, suggesting that AtMnd1 is required for DSB repair in somatic cells (Domenichini et al., 2006). Hence, MND1 may be an interesting drug target to sensitize somatic cancers to DSB-inducing therapy.

### SPO11

The human *SP O 11* gene is located in chromosome 20q13.2-13.3, a region that is amplified in multiple breast cancers and associated with genomic instability (Tanner et al., 1994; Courjal et al., 1996; Collins et al., 1998). However, there are limited studies to date that have investigated the potential role of SPO11 in carcinogenesis. The aberrant expression of *SP O 11* has been reported in patients samples of melanoma (Koslowski et al., 2002), colorectal cancer (Eldai et al., 2013), cervical cancer (Koslowski et al., 2002) as well as in Acute Myeloid Leukemia (AML) (Atanackovic et al., 2011), CTCL (Litvinov et al., 2014) and lung cancer (Koslowski et al., 2002) cell lines. High-resolution cytogenetic microarray data of 15 tumor-normal paired colorectal cancer samples revealed a gain in chromosome copy number of the *SP O 11* gene (Eldai et al., 2013). Increased *SP O 11* expression was also detected in patients with CTCL compared to expression in normal skin and benign inflammatory dermatoses (Litvinov et al., 2014). Based on the function of SPO11 in the induction of DSBs, it would be interesting to investigate whether its expression contributes to the genomic instability by promoting translocations, insertions and deletions.

### PRDM9

*PRDM9* is recurrently mutated in head and neck squamous cell carcinoma (Stransky et al., 2011), and an excess of rare *PRDM9* alleles has been reported in aneuploid and infant B-cell precursor acute lymphoblastic leukemia patients (Hussin et al., 2013). Based on its function, altered *PRDM9* expression could create vulnerable DNA regions that are favorable to the formation of chromosomic lesions. Indeed, new evidence has recently emerged to suggest a link between PRDM9-driven meiotic recombination hotspots and genomic instability (Houle et al., 2018; Kaiser and Semple, 2018). In a study where *PRDM9* expression was analyzed in 1879 cancer samples, *PRDM9* was unexpectedly found to be expressed in 20% of these tumors. Intriguingly, *PRDM9* expression correlated with areas of chromosomal instability and in samples with aberrant *PRDM9* expression, structural variant breakpoints frequently neighbor the DNA motif recognized by PRDM9 (Houle et al., 2018). This might suggest that PRDM9 generates chromatin regions that become more fragile and could favor genomic instability. All this evidence has raised the interest for targeting meiotic genes that are aberrantly expressed in somatic cancer cells. In a recent study, Allali-Hassani and colleagues reported the discovery of a potent and selective PRDM9 inhibitor (MRK-740) (Allali-Hassani et al., 2019). In HEK293T cells, MRK-740 specifically and directly inhibited PRDM9 catalytic activity on chromatin, reducing H3K4 methylation at intragenic and intergenic target sites. However, MRK-740 did not reveal any significant effect on proliferation of several cancer cell lines tested, indicating that at least for the cell lines tested their proliferation was not PRDM9-dependent (Allali-Hassani et al., 2019).

### DMC1

Similarly to other genes involved in meiotic recombination, DMC1 was found to be ectopically expressed in various cancer cell lines including cervical (Erenpreisa et al., 2009), colon (Ianzini et al., 2009), breast (Salmina et al., 2019), glioblastoma (Rivera et al., 2015) and lymphoma cancer cell lines (Kalejs et al., 2006) as well as in CTCL biopsy samples (Gantchev et al., 2020). Interestingly, the upregulation of DMC1 was reported in a number of studies to drive the resistance of cancer cells to various cytotoxic and genotoxic agents (Kalejs et al., 2006; Erenpreisa et al., 2009; Ianzini et al., 2009; Rivera et al., 2015; Salmina et al., 2019). When challenged with high doses of ionizing radiation, tumor cells can escape cell death by transient endopolyploidisation (Illidge, 2000). While most of these polyploid cells will undergo cell death following aberrant mitosis (mitotic catastrophe), some will undergo genome reduction giving rise to viable tumor cells with reduced ploidy that can resume the mitotic cell cycle and are resistant to the treatment (Illidge, 2000). Experiments conducted with the large-scale digital cell analysis system, show that meiosis-specific genes such as DMC1, are expressed in the polyploid cells during depolyploidization allowing them to escape radiation-induced cell death (Ianzini et al., 2009). The study suggests that tumor cells might take advantage of the temporary change from a pro-mitotic to a pro-meiotic division regimen to facilitate depolyploidization and restore the proliferative state of the tumor cell population (Ianzini et al., 2009). A few years later, another study investigated the aberrant activity of DMC1 in glioma and showed that loss of DMC1 inhibited the activation of the DNA damage response and increased radiosensitivity. Furthermore, loss of DMC1 reduced tumor growth and prolonged survival *in vivo* (Rivera et al., 2015). These data suggest that the activation of meiotic repair genes in neoplastic cells selectively provides tumor cells with a repair mechanism to evade cell death caused by DNA damage, while at the same timeincreasing genetic diversity to drive clonal evolution (Rivera et al., 2015).

### MEIOB

Analysis of multiple independent transcriptome databases containing both normal and tumor samples, identified the aberrant activation of MEIOB in lung adenocarcinomas (Wang et al., 2016). In the same study its meiotic partner, *SPATA22,* was also found to be aberrantly activated and co-expressed with *MEIOB*. Expression of MEIOB was also greatly enhanced in several lung cancer cell lines after treatment with the DNA methylation inhibitor 5-Aza-2′-deoxycytidine, known to induce the expression of certain meiotic genes by the demethylation of promoter CpG islands (De Smet et al., 1999). More recently, MEIOB aberrant expression was reported *in vitro* and *in vivo* models for TNBCs as well as in patients, where it correlated with poor survival (Gu et al., 2021). The authors of the study showed that MEIOB significantly promoted the proliferation of TNBC cells as well as DSBs repair. However, in contrast to its function in meiosis, MEIOB expression mediated homologous recombination deficiency (HRD) through the activation of polyADP-ribose polymerase (PARP). Furthermore, MEIOB was shown to confer sensitivity to PARP inhibitors *in vitro*, as well as in a PDX model of TNBC (Gu et al., 2021). Together this suggests that MEIOB expression could be useful as a predictive biomarker of PARP inhibitor response in TNBC.

### Genes of the Cohesin Complex

Consistent with roles in chromosome segregation and regulation of gene expression, aberrant expression and malfunctioning of cohesins is expected to be associated with cancer development (Losada, 2014). Indeed, several studies reported that meiosis-specific cohesins are aberrantly expressed in different types of somatic cancers.

#### STAG3

As most meiosis-specific genes, STAG3 is silenced in somatic cells by methylation of histone H3 on lysines 9 and 27 (Storre et al., 2005). However, reactivation of the cancer testis antigen STAG3 has been reported in cancers. For instance, mutations on the *STAG3* gene in cases of colorectal cancers have been identified (Barber et al., 2008). While it has still to be clarified whether the aneuploidy and tumorigenesis observed in these cancers are due to altered gene expression or due to chromosome mis-segregation (or both), the authors suggest that these mutations may lead to chromosome instability. Aberrant expression of STAG3 was also reported in patient-derived lymphocytes isolated from a CTCL patient as well as in skin biopsy samples from Sézary Syndrome patient (Tsang et al., 2018; Gantchev et al., 2020). Microarray analysis associated *STAG3* gene expression with tumorigenicity in ovarian cancer cell lines (Notaridou et al., 2011) while another study reported that multiple meiotic genes, including *STAG3*, are aberrantly activated during mitotic catastrophe in lymphoma cells after irradiation and may mediate chromosomal mis-segregation and genome reduction (Kalejs et al., 2006).

#### REC8

One of the first indications of a role for REC8 in cancer progression comes from a study that revealed REC8 upregulation in *Tp53*-mutated lymphoma cells after irradiation. REC8-augmented expression induced mitotic catastrophe and the generation of endopolyploid tumor cells (Kalejs et al., 2006). Similar findings were reported in additional endopolyploid p53-deficient tumor cells, where REC8 upregulation upon irradiation induced pseudomeiotic chromosome segregation events that enabled them to survive genotoxic treatment (Erenpreisa et al., 2009). A few years later, the work of Grewal et al. in fission yeast significantly contributed to the understanding of the mechanistic role of REC8 in cancer progression (Folco et al., 2017). The authors found that upregulation of REC8 expression was caused by the dysregulation of the Mmi1 pathway, which plays a crucial role in suppressing meiotic genes during mitotic proliferation (Harigaya et al., 2006). This causes high levels of chromosome mis-segregation events in mitotically dividing diploid cells, including high levels of uniparent disomy (UPD), a phenomenon that is linked to congenital disorders (Mobasheri et al., 2007)and various cancers (Tuna et al., 2009; Andersen and Petes, 2012), where it can drive loss of heterozygosity. Strikingly, REC8 overexpression in mitotically dividing diploid cells was sufficient to induce UPD, suggesting that the expression of a single meiotic cohesin gene is enough to promote meiosis-like reductional segregation of homologues in mitotic cells. In contrast to other meiotic genes, reactivation of REC8 in mitotic cells was also shown to play a tumor suppressor role in certain cancer cell lines, such us gastric cancer cells where induced overexpression of REC8 inhibited cell proliferation, invasion and migration (Yu et al., 2017; Zhao et al., 2018). However, the role of REC8 as a tumor suppressor remains elusive and further studies are needed to decipher how reactivation of a cohesin protein could protect cells from cancer progression.

### Genes of the Synaptonemal Complex

The formation of the SC is mediated by proteinaceous axial structures, which include the central SYCP1 and the two lateral SYCP2 and SYCP3 components. Remarkably, re-expression of synaptonemal complex genes has been implicated in cancer to modulate the level of genome integrity (Gantchev et al., 2020; Hosoya and Miyagawa, 2021).

#### SYCP1

Aberrant expression of SYCP1 was first reported in melanoma, breast cancer, glioma, stomach cancer, NSCLC and renal carcinoma (Tureci et al., 1998). Subsequently, elevated SYPC1 expression was also reported in other types of tumors and cancer cell lines including gastric (Mashino et al., 2001), hepatocellular (Chen et al., 2001), pancreatic adenocarcinomas (Kubuschok et al., 2004), head and neck squamous cell carcinoma (Atanackovic et al., 2006), meningiomas, astrocytomas and oligodendrogliomas (Sahin et al., 2000), medulloblastomas (Oba-Shinjo et al., 2008) and testicular germ cell tumors (Zhang et al., 2005). SYCP1 expression was also detected in various hematological malignancies such as myelomas, acute lymphatic leukemia (AML), chronic myeloid leukemias (Lim et al., 1999), acute lymphocytic leukemias (Niemeyer et al., 2003), chronic lymphocytic leukemia, B-Cell lymphomas, Burkitt’s lymphomas, lymphoblastic lymphomas (Xie et al., 2003) and non-hodgkin’s lymphomas (Huang et al., 2002). Despite the expression of SYCP1 in a vast variety of tumors, there is currently no solid evidence describing the consequence of ectopic expression in somatic cancer cells or the underlying mechanism of action.

#### SYCP3

SYPC3 expression has also been documented in various cancers, including NSCLC (Kitano et al., 2017), acute lymphoblastic leukemia (Niemeyer et al., 2003), breast cancers, brain, gastrointestinal, skin tumors (Mobasheri et al., 2007) and cervical cancers (Hanbyoul Cho et al., 2014). It was reported that SYCP3 expression can be induced in the colorectal carcinoma cell line DLD1 after treatment with the demethylating agent 5-azacytidine, indicating that SYCP3 expression in mitotic cells is regulated by a demethylation-dependent process, similarly to other meiotic genes (Hosoya et al., 2012). The clinical relevance of SYCP3 expression was described in cervical cancer and NSCLC. Cho et al., examined SYCP3 expression in tumor specimens from 181 cervical cancer and 400 cervical intraepithelial neoplasia (CIN) patients by immunohistochemistry and analyzed the correlation between SYCP3 expression and clinicopathologic factors or survival. High expression of SYCP3 was significantly associated with late stage and high grade. At a molecular level, SYCP3 expression positively correlated with pAKT protein levels, suggesting that SYCP3 role in carcinogenesis may be mediated by an activated AKT signaling (Hanbyoul Cho et al., 2014). In NSCLC, there are two studies describing the clinical relevance of SYCP3 expression. Immunohistochemical and tissue microarray analysis of NSCLC patient samples revealed high cytoplasmic SYCP3 expression, which correlates with early stage NSCLC, lymph node metastasis, pleural invasion and poor survival (Chung et al., 2013). Consistent with these data, increased SYCP3 expression was also detected in another immunohistochemical analysis in NSCLC cases with lymph node metastasis (Kitano et al., 2017). In this study, SYPC3 expression positively correlated with VEGF-C and VEGF-D expression, which are both involved in NSCLC lymphangiogenesis and metastatic spread to lymph nodes (Kitano et al., 2017). Mechanistically, SYCP3 expression outside the meiotic context has been shown to disrupt the activity of the tumor-suppressing recombination regulator BRCA2 (Hosoya et al., 2012). In SYCP3-expressing somatic cells, the BRCA2-mediated recruitment of RAD51 to the break site is in fact inhibited, resulting in defective sister-chromatid recombination. The authors of the study further show that expression of SYCP3 inhibits homologous recombination, inducing hypersensitivity to DNA-damaging agents such as PARP inhibitors and chromosomal instability. These findings highlight a new mechanism for genomic instability and extend the range of PARP-inhibitor sensitive tumors to those expressing SYCP3 (Hosoya et al., 2012).

## Summary

From these studies, it emerges that the ectopic activation of meiotic genes is detected in a wide variety of cancers, where it drives genomic instability and cancer progression. Even if cancer cells are not dependent on these genes for normal growth, they may become essential in tumors (but not in healthy tissues) to tackle endogenous DNA damage or DNA lesions induced by anticancer therapies. Indeed, most of the meiotic genes that are aberrantly expressed in cancer cells have a direct or indirect effect on pathways that are responsible for the repair of the DSBs induced by anticancer therapies. Examples include the HORMAD1/2, MND1, MEIOB and SYCP3 genes, which directly influence the HR activity of cancer cells. Their loss may induce sensitivity to agents that put more pressure on a functional HR pathway, such as PARP inhibitors. Other genes, including DMC1, STAG3 and REC8, allow somatic cancer cells to escape radiation-induced cell death without directly affecting the intracellular DNA repair pathways. Instead, they appear to promote meiosis-like reductional segregation of homologues in polyploid cells and thereby restore the proliferative state of the tumor cell population. For SPO11 and PRDM9, which induce DNA strand breaks and create crossover events in cancer cells, one can speculate that their activation in cancer cells drives genomic instability and might therefore increase the sensitivity of these cells to DNA-damaging agents.

The expression of meiotic genes in somatic cells appears to provide an evolutionary advantage for cancer initiation and progression. Such re-expression occurs via different mechanisms, including gain in copy number, increased expression following a genotoxic stress, and most frequently, via demethylation of meiotic gene promoters. In addition to promoting genomic instability, the activation of germ cell genes in mitotic cells influences how cells handle genomic instability.

While the re-expression of meiosis-specific genes promotes cancer progression, it may provide a new vulnerability that can be exploited therapeutically. As ectopic expression of meiotic genes has been shown to affect the response of tumor cells to anticancer therapies, it might be used as a predictive biomarker of therapy response and thus guide treatments’ decision in the clinic. Further, meiotic genes represent promising candidate targets for cancer immunotherapy with little risk of side effects, due to high tumor specificity and immunogenicity. Since germ cells in adults lack HLA-class I molecules and cannot present the antigens to T cells, meiotic genes expressed in cancer cells have the capacity to promote immune responses that are strictly cancer specific. There are currently two immunotherapy strategies that are being tested in clinical settings, which exploit meiotic genes as cancer antigens: adoptive transfer, where recombinant T-cell receptors specific for cancer antigen epitopes are inserted into patient T cells and transferred back to patients, and vaccination, which stimulates the patient’s intrinsic immune response to cancer antigens thanks to the use of immunogenic peptides (Gjerstorff et al., 2015). The therapeutic function of these two approaches is currently being tested in a variety of clinical settings and recent clinical trials have provided encouraging results (Gjerstorff et al., 2015).

We therefore think that studying the role of meiotic genes in somatic cancers is an interesting area to further explore, particularly in the context of DSB repair. We may also find out that several of the genes that we link to meiosis-specific exchange of genetic information actually have an additional and thus far unknown role in homology-directed DNA repair in somatic cells, even in non-transformed ones. It may not be remnants of embryonic tissue, but rather remnant DSB repair pathways that are reactivated to promote cancer growth.
